# Bridging global gaps in somatoform disorder

**DOI:** 10.1097/MS9.0000000000004882

**Published:** 2026-03-31

**Authors:** Aneeq Mahmood Khan, Ayesha Mustafa, Aqsa Raza, Muhammad Sarim Azad Khan, Muhammad Shahmeer Khan, Mahnoor Fatima

**Affiliations:** a Department of Medicine, Sargodha Medical College, Sargodha, Pakistan; b Department of Medicine, Fatima Jinnah Medical University, Lahore, Pakistan; c Department of Medicine, Khyber Girls Medical College, Peshawar, Pakistan; d Department of Medicine, Combined Military Hospital Lahore Medical College, Lahore, Pakistan; e Department of Medicine, Ayub Medical College, Abbottabad, Pakistan; f Department of Medicine, Shaheed Ziaur Rahman Medical College, Rajshahi Medical University, Bogura Bangladesh

This letter comments on the recent study by Siig *et al*^[^1^]^, which provides a pooled global prevalence estimate for somatoform disorders of ~4.6%, with higher rates in women (7.7%) than men (2.8%) and substantial years lived with disability (662.4 YLDs per 100 000), signaling a major disability burden. While this work marks progress in measurement, critical gaps in geographic representation and diagnostic consistency persist, limiting accurate global understanding and interventions^[^1^]^.

Available evidence is dominated by surveys from high-income regions with insufficient data from low- and middle-income countries (LMICs), where primary care often relies on symptom presentation that may mask somatic disorders. Diagnostic heterogeneity further complicates comparisons. DSM-5 somatic symptom disorder (SSD) requires ≥1 persistent somatic symptom plus excessive thoughts, feelings, or behaviors. In contrast, ICD-11 bodily distress disorder (BDD) focuses on multiple bodily symptoms and their functional impact without specifying symptom count. Large surveys report a prevalence ranging from 2.0% for BDD to 3.5% for SSD, resulting in a pooled prevalence of 4.1% across diverse populations^[^2^]^. Notably, women more frequently report pain, gastrointestinal, and fatigue-related symptoms, contributing to observed prevalence ratios (2.7:1) even after adjusting for comorbidities^[^3^]^.

These knowledge and diagnostic gaps matter for clinical practice, policy, and research. Insufficient LMIC data and inconsistent diagnostic definitions undermine accurate global burden estimates, early recognition, and cross-national comparability, ultimately affecting resource allocation and care planning^[^1^]^. The most striking gender differences involve a higher prevalence of pain and fatigue symptoms in women, suggesting biological, psychological, and sociocultural influences^[^1,3^]^, which may bias both research interpretation and clinical detection.

To address these limitations, several coordinated actions are essential. First, adoption of standardized, harmonized diagnostic frameworks that align with DSM-5 and ICD-11 approaches will improve cross-study comparability and surveillance^[^4^]^. However, such harmonization efforts should be implemented cautiously, as they may risk oversimplifying culturally specific presentations of somatic symptoms in diverse populations. Second, investments in large-scale epidemiological studies in LMICs are required to capture diverse presentations and inform equitable global health policy. Feasible implementation strategies could include multisite collaborative designs, integration with existing national health surveys, or leveraging digital tools for broader reach. Third, gender-specific research designs should be embedded to clarify biological versus social determinants of somatic symptom patterns^[^5^]^. Fourth, routine inclusion of somatoform and related disorders into global surveillance initiatives, including future cycles of the Global Burden of Disease Study, can elevate awareness, inform funding decisions, and integrate evidence into mental health planning at national and international levels^[^1,2^]^.

In conclusion, notwithstanding advances in prevalence estimation, enduring data limitations and diagnostic heterogeneity continue to obscure the true global burden of somatoform disorders. The adoption of harmonized diagnostic frameworks, more inclusive epidemiological methodologies, and gender-responsive research agendas is essential to achieve accurate burden quantification, reinforce mental health system planning, and inform effective clinical interventions and evidence-based policy development. This letter is in line with the TITAN Guidelines on the need for transparency in AI use in healthcare^[^6^]^.

## Data Availability

Not applicable to this study.
